# NMR structure of the water soluble Aβ_17–34_ peptide

**DOI:** 10.1042/BSR20140094

**Published:** 2014-11-24

**Authors:** Genadiy Fonar, Abraham O. Samson

**Affiliations:** *Faculty of Medicine in the Galilee, Bar-Ilan University, Safed 14300, Israel

**Keywords:** Alzheimer’s disease, amyloid beta, amyloid β, Aβ17–34, NMR, soluble peptide, Aβ, amyloid β, AChR, acetylcholine receptor, APP, amyloid precursor protein, HFIP, hexafluoroisopropanol, nAChR, nicotinic acetylcholine, TFE, trifluoroethanol receptor

## Abstract

Alzheimer's disease is the most common neurodegenerative disorder in the world. Its most significant symptoms are memory loss and decrease in cognition. Alzheimer's disease is characterized by aggregation of two proteins in the brain namely Aβ (amyloid β) and tau. Recent evidence suggests that the interaction of soluble Aβ with nAChR (nicotinic acetylcholine receptors) contributes to disease progression. In this study, we determine the NMR structure of an Aβ_17–34_ peptide solubilized by the addition of two glutamic acids at each terminus. Our results indicate that the Aβ peptide adopts an α-helical structure for residues 19–26 and 28–33. The α-helical structure is broken around residues S26, N27 and K28, which form a kink in the helical conformation. This α-helix was not described earlier in an aqueous solution without organic solvents, and at physiological conditions (pH 7). These data are in agreement with Aβ adopting an α-helical conformation in the membrane before polymerizing into amyloid β-sheets and provide insight into the intermediate state of Aβ in Alzheimer's disease.

## INTRODUCTION

Alzheimer's disease is widely characterized by amyloid peptide deposits inside brain tissues [1]. These deposits, also called amyloid plaques, present one of the most characteristic feature of the disease and can be seen in different brain regions [2]. Amyloid plaques consist of Aβ (amyloid β) peptides that become aggregated [3–5]. The secondary structure of aggregated Aβ is mostly β-sheet [6–8] and results from enzymatic cleavage of the APP (amyloid precursor protein) [9,10] by α, β and γ-secretases [11]). When APP is cleaved by α-secretase, the resulting product is a protein named soluble APPα, while cleavage with the two other enzymes, β and γ-secretases, results in the creation of a 40 or a 42 amino acids long peptide–namely Aβ [11]. Mutations in any one of the secretases leads to increased production of Aβ, and a higher susceptibility for Alzheimer's disease [12].

Current structure determination techniques include NMR, X-ray crystallography and cryo-EM (cryoelectron microscopy). Although X-ray crystallography is the most accurate technique for determining the structure of large proteins, NMR is considered more suitable for elucidating the structure of short peptides at physiological conditions [13]. Several studies have attempted to determine the structure of the Aβ peptide using NMR, but disconcertingly most of them had to use hydrophobic solvents such as TFE (trifluoroethanol) [14–16] and HFIP (hexafluoroisopropanol) [17] to increase solubility. Adding TFE and HFIP to Aβ can induce conformational changes and impose an artificial α-helical structure [18], thus providing the researchers with biased structural information. Remarkably, one attempt to determine the NMR structure without TFE at physiological conditions, revealed Aβ to adopt a 3_10_-helical structure incongruent with previous α-helical observations [19]. Finally, another NMR study used micelles to determine the structure of Aβ_10–35_ and found it to possess two α-helical regions within residues 13–23 and 30–35 [20].

In this study, we determine the structure of a soluble Aβ peptide corresponding to residues 17–34 which comprises the nucleation site of Aβ using NMR spectroscopy at physiological conditions. We find it to adopt a partial α-helical conformation in agreement with bioinformatic secondary structure predictions. This finding sheds light on the Aβ conformations in Alzheimer's disease and provides a snapshot of the intermediate structure prior to conversion into amyloid plaques.

## EXPERIMENTAL

### Aβ peptides

Aβ peptides corresponding to residues 17–34 were purchased and purified by the purveyor to a purity level of >99.9%, using HPLC. These peptides contained two glutamate or arginine residues at the N- and C-termini for increased solubility. The full amino acid sequence of the purchased Aβ was EELVFFAEDVGSNKGAIIGLEE (Peptide 2.0 Inc. Company) and RRLVFFAEDVGSNKGAIIGLRR (Sigma-Aldrich). The peptide mass was verified using MALDI-TOF mass spectroscopy. To dissolve the peptides, phosphate and acetate aqueous buffers were prepared. None of the buffers contained hydrophobic solvents known to solubilize and stabilize α-helices (i.e. TFE or HFPI) or surfactants known to dissolve hydrophobic peptides (i.e. SDS). Henceforth, the Aβ_17–34_ peptide refers to the peptide flanked by two glutamates.

### NMR sample preparation

To prepare a sample for NMR measurements, 1 mg of Aβ_17–34_ peptide was dissolved in 300 μl of 50 mM phosphate buffer (pH 7). To this solution, 15 μl of D_2_O (Sigma-Aldrich) and 1 μl of sodium azide (100 μM) (Sigma-Aldrich) were added to a final concentration of 50 mM PO_4_, 5% D_2_O and 0.3 μM NaN_3_. This NMR sample was pipetted into a Shigemi tube (Shigemi Corporation) and sealed with a plunger.

### NMR experiments

^1^H-NMR experiments were performed on a 600 MHz Bruker NMR spectrometer at 278 K. After calibration, 2D TOCSY and ROESY spectra were measured [21–23]) with 2048 and 1024 points in the F2 and F1 dimensions, respectively and 160 scans. The WATERGATE pulse sequence was used in order to suppress the water signal [24,25]. Mixing time for ROESY experiment was set to 400 ms for proper magnetization transfer. Spectral processing was carried out using Bruker TopSpin (version 3.2) software. Spectrum and chemical shift assignment were carried out using the sequential assignment technique developed by Kurt Wüthrich [13].

### Structure determination

Structure determination was performed using the Crystallography and NMR system (CNS, version1.3) software suite [26]. Structures were calculated with the distance geometry and simulated annealing protocols followed by energy minimization using distance and dihedral angle constraints. Dihedral angle constraints were derived from the ^3^J_HNHα_-couplings measured in the TOCSY spectrum processed with 4096 points in the F2 dimension. Distance constrains were calculated from cross-peak height in the ROESY spectrum.

## RESULTS

### Peptide solubility

To solubilize Aβ, several approaches were taken. One approach was to add two arginines at the N- and C-termini. This addition increased the theoretical peptide pI from 4.37 (without arginines) to 11.54, but did not improve solubility in acetate and phosphate buffers at pH 1, 7 and 10. Another approach was to add two glutamic acids at the N- and C-termini. This addition decreased the theoretical peptide pI from 4.37 (without glutamates) to 3.83, and improved solubility drastically. The glutamic acid peptides were soluble in both 50 mM acetate and 50 mM phosphate buffers at pH 7 but not in water.

### NMR sample preparation

NMR samples containing 1 mg of Aβ_17–34_ peptide dissolved in 300 μl of phosphate buffer displayed a pH of 7 (uncorrected for the isotope effect). The NMR samples did not show visible sedimentation or aggregation following 5 min centrifugation at 14000 Rev. per min at 278 K.

### NMR measurements

**Figure 1:**
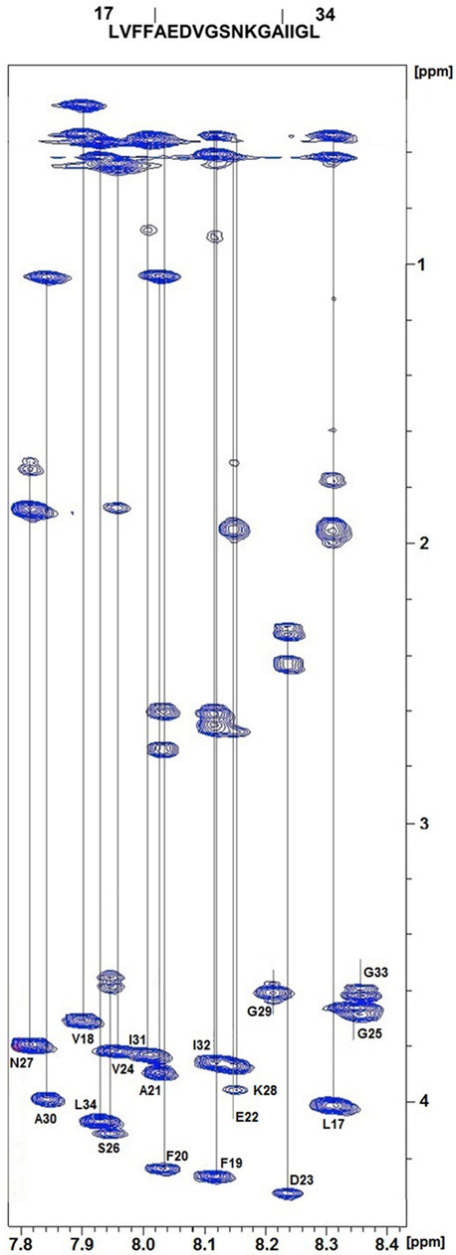
Fingerprint region of TOCSY spectrum of Aβ_17–34_ peptide Each line represents one amino acid system.

The chemical shifts of all protons of the Aβ_17–34_ peptide were identified in the TOCSY spectrum using Wüthrichs sequential assignment technique. Figure 1 shows the TOSCY spectrum of this peptide with its 18 amino acid systems. The four glutamic acid residues, two at the C-terminus and two at the N-terminus were not detected due to their high flexibility. The spectrum showed one system per amino acid and so was suggestive of a single conformation.

**Figure 2:**
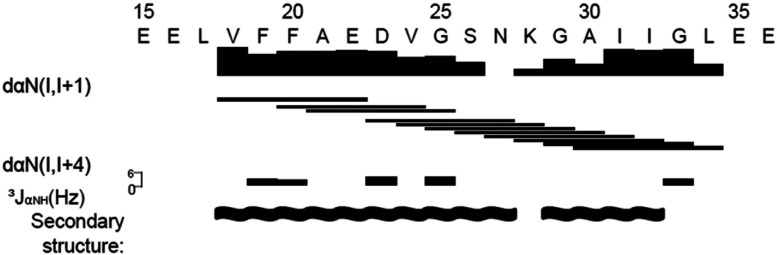
Summary of NMR data used for the sequential assignment and secondary structure determination of Aβ_17–34_ peptide The data were obtained from the ROESY spectrum recorded at 278 K and pH 7 with 400 ms mixing time. Line thickness indicates the relative cross-peak height of the sequential NOE connectivities.

The ROESY spectrum (not shown) displayed intermediate range Hα(*i*)/HN(*i+*4) cross-peaks between the following amino acid pairs: V18-E22, F20-V24, A21-G25, D23-N27, V24-K28, G25-G29, S26-A30, N27-I31, K28-I32, G29-G33 and A30–L34 (summarized in Figure 2). These cross-peaks were indicative of an α-helix conformation. Additional data supporting the α-helical structure include ^3^J_HNHα_-couplings of residues F19, F20, D23, G25 and G33 with values of 6 Hz or less (Figure 2). Also shown in Figure 2 is a summary of the Hα(*i*), HN(*i+*1) connectivities used for the sequential assignment. Interestingly, no HN-Hα cross-peak was observed between residues N27 and K28, suggesting a flexible random coil (or kink) in the helix around residues N27 and K28.

### NMR structure

**Figure 3:**
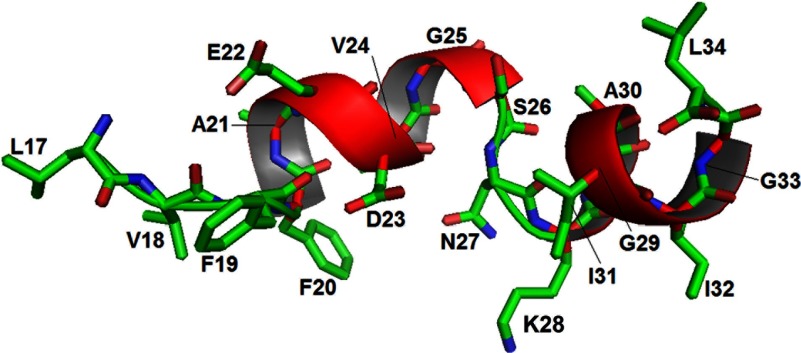
Average NMR structure of the Aβ_17–34_ peptide Shown is a ribbon diagram and stick representation of the Aβ_17–34_ structure. Notice the two α-helices formed by segments 19–26 and 28–33 with a break around residues S26, N27 and K28.

The lowest energy NMR structures of the soluble Aβ_17–34_ peptide were found to adopt an α-helix conformation for most of the residues, with a break around S26, N27 and K28 in six out of ten ensemble structures. Figure 3 shows the average structure of the Aβ_17–34_ peptide. The α-helix spans residues 19–26 and 28–33 with a pronounced kink around residues S26, N27 and K28. Interestingly, the α-helix of residues 23–26 and 28–33 was not previously described. Please note that residues 17–23 and 33–34 did not adopt helical conformation probably due them being terminal residues. Table 1 illustrates the statistical data of the final set of ten structures calculated using CNS. Distance constraints from the ROESY spectrum originated mainly from the fingerprint region. Torsion angle constraints were measured in the TOCSY spectrum with increased processing resolution and identified for residues F19, F20, D23, G25 and G33.

**Table 1:** NMR constrains and structural statistics for 10 Aβ_17–34_ peptide structures

Measurements	
NMR distance constrains	
Total constrains	86
Medium range (|*i*–*j*|<4)	11
Torsion angle constraints (φ)	5
Mean RMSD values	
Backbone atoms	1.48
Backbone atoms of α-helix	0.28

Table 2 shows a Chou–Fasman secondary structure prediction of the Aβ_1–40_ peptide. [27]. The secondary structure was also predicted using more modern servers (NetsurfP [28], Jpred [29], I-TASSER [30]); however, the confidence level in these was not high. It is interesting to note that the Aβ peptide shows a high propensity for both α and β secondary structure. This tendency is an important hallmark in secondary structure conversion of the Aβ peptide. Our α-helical structure is in agreement with the Chou–Fasman prediction and note that the kink observed around residues S26, and N27 coincides with residues G25 (α-helix propensity of 0.43), S26 (α-helix propensity of 0.57) and N27 (α-helix propensity of 0.76), which possess the lowest α-helix propensities of all amino acids, except proline [31].

**Table 2:** Chou–Fasman secondary structure prediction of Aβ_1–40_

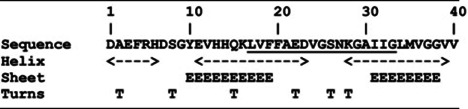

## DISCUSSION

Several studies have shown that soluble Aβ and Alzheimer's disease are intertwined and that soluble Aβ peptide contributes to disease development and progression [32]. Nevertheless, many important details about soluble Aβ including its structure are missing, biased or contradicting.

Several studies found that Aβ peptides possess helical or random coil structures under non-physiological conditions. One NMR study found Aβ_1–40_ to adopt an 3_10_-helix in residues 13–23 in aqueous environment (PDB ID 2LFM) [19]. Another NMR study found Aβ_10–35_ to adopt an α-helix in residues 13–23 and 30–35 inside SDS micelles [20]. However, at pH 7.3 Aβ_10–35_ is unstructured and adopts a compact random-coil conformation. In another study, the NMR structure of Aβ_1–42_ in a solution containing HFPI/H_2_O 30:70 adopts an α-helical conformation in residues 8–25 and 28–38 (PDB ID 1Z0Q) [17]. This finding is similar to ours; however we did not use solubilizing solvents capable of altering protein conformation.

In addition, Aβ was also found to adopt β-hairpin and β-sheet conformations. In one FTIR (Fourier-transform infrared) study, Aβ oligomers were found to adopt an antiparallel β-sheet structure [33]. Another study suggested that Aβ_12–42_ adopts an antiparallel β-sheet conformation with a turn at residues 25–28 [34]. Finally, a third NMR study of Aβ_1–40_ in complex with a phage-display selected antibody, found that residues 17–36 adopt a β-hairpin conformation with a turn composed of residues 23–30 (PDB ID 2OTK) [35].

Our findings suggest that the α-helix is longer than previously observed [19] and it is in agreement with secondary structure predictions (Table 2). We did not observe the α-helix described in residues L17 and V18 (Table 2), probably because the peptide is truncated at residue 17. Also, we did not observe an α-helical conformation for residue L34 because the peptide is truncated here too.

### Interaction with nAChR (nicotinic acetylcholine receptor)

Since the first report of Aβ interacting with the nAChR was published [36] several studies attempted to determine the binding mode ([37] and references therein). Before binding the AChR (acetylcholine receptor), Aβ possesses a helical structure with a kink around residue K28. As was reported by us previously [37], K28 is an important anchor point for the amyloid in AChR. A kink around this residue would indeed facilitate anchoring and interaction between the Aβ and AChR. One could hypothesize that binding to AChR condemns it to undergo structural change form helix to β-sheet inside the binding pocket and leaves the peptide in β-sheet form, allowing it to form fibrils and plaques later. In this study, we show the intermediate soluble state of the Aβ, which could facilitate binding to the AChR.

Conformational transition

**Figure 4:**
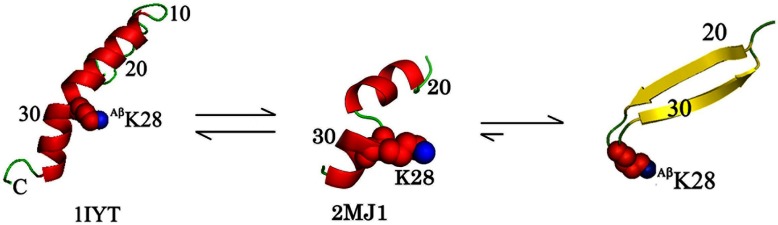
Proposed model of Aβ structural changes Shown from left to right is a proposed mechanism of Aβ transition from α-helix to β-sheet. Shown on the left is the α-helical solution structure of Aβ_1–42_ in an membrane-like environment (PDB ID 1IYT) [15]. Shown in the middle is our average NMR structure of the Aβ_17–34_ peptide (PDB ID 2MJ1). Shown on the right is the β-hairpin conformation of the Aβ peptide [37]).

Interestingly, based on secondary structure propensities, the Aβ peptide shows a dual preference for α-helix and for β-sheet (Table 2). This double preference may be attributed to the environment, and Aβ embedded in membranes preferentially adopts an α-helical conformation. Contrarily, in aqueous environment the equilibrium between these states is pushed towards a β-sheet structure, and once this occurs, aggregation and sedimentation follow. In this study, we show the delicate intermediate state of the Aβ peptide solubilized by four glutamate residues. Using NMR, we show that Aβ peptide possesses a stable α-helical structure within residues F20–S26 and K28–G33 with a break around S26–N27–K28. The helical structure was not previously reported in an aqueous environment in the absence of organic solvents, and only a 3_10_-helix was observed. Another important finding of this research is a possible explanation regarding amyloids structural change from α-helix to β-sheet. Based on our structure, the existence of a kink in the middle of the peptide, which breaks it into two helices allows the peptide to undergo structural changes from α-helix to β-sheet once the two halves come close to one another (Figure 4). Where it not for the solubilizing glutamate residues, then Aβ17–34 would aggregate immediately upon leaving the membrane.

### Conclusions

This study sheds light on the soluble structure of Aβ and broadens our biomolecular understanding of Alzheimer's diseases. To the best of our knowledge, this study is the first to report an α-helix in Aβ in aqueous environment and at physiological conditions. The structure provides an important snapshot of the intermediate state between the pure α-helix and β-sheet conformations, and contributes to our understanding of the conformational transition of Aβ in Alzheimer's disease.

## References

[1] Alzheimer A., Stelzmann R. A., Schnitzlein H. N., Murtagh F. R. (1995). An English translation of Alzheimer's 1907 paper, ‘Uber eine eigenartige Erkankung der Hirnrinde’. Clin. Anat..

[2] Hyman B. T., Marzloff K., Arriagada P. V. (1993). The lack of accumulation of senile plaques or amyloid burden in Alzheimer's disease suggests a dynamic balance between amyloid deposition and resolution. J. Neuropathol. Exp. Neurol..

[3] Masters C. L., Beyreuther K. (2005). Neurodegenerative Diseases.

[4] Bruggink K. A., Müller M., Kuiperij H. B., Verbeek M. M. (2012). Methods for analysis of amyloid-β aggregates. J. Alzheimers Dis..

[5] Etienne M. A., Edwin N. J., Aucoin J. P., Russo P. S., McCarley R. L., Hammer R. P. (2007). Beta-amyloid protein aggregation. Methods Mol. Biol..

[6] Sunde M., Serpell L. C., Bartlam M., Fraser P. E., Pepys M. B., Blake C. C. (1997). Common core structure of amyloid fibrils by synchrotron X-ray diffraction. J. Mol. Biol..

[7] Lührs T., Ritter C., Adrian M., Riek-Loher D., Bohrmann B., Döbeli H., Schubert D., Riek R. (2005). 3D structure of Alzheimer's amyloid-beta (1–42) fibrils. Proc. Natl. Acad. Sci. U. S. A..

[8] Scheidt H. A., Huster D., Rothemund I., Morgado S. (2012). Dynamics of amyloid fibrils revealed by solid-state NMR. J. Biol. Chem..

[9] O’Brien R. J., Wong P. C. (2011). Amyloid precursor protein processing and Alzheimer's disease. Annu. Rev. Neurosci..

[10] Zhang Y., Thompson R., Zhang H., Xu H. (2011). APP processing in Alzheimer's disease. Mol. Brain..

[11] Epis R. (2012). Alpha, beta-and gamma-secretases in Alzheimer's disease. Front. Biosci..

[12] Hardy J., Allsop D. (1991). Amyloid deposition as the central event in the aetiology of Alzheimer's disease. Trends Pharmacol. Sci..

[13] Wuthrich K. (1986). NMR of Proteins and Nucleic Acids.

[14] D’Ursi A. M., Armenante M. R., Guerrini R., Salvadori S., Sorrentino G., Picone D. (2002). Solution structure of amyloid β-peptide (25–35) in different media. J. Med. Chem..

[15] Crescenzi O., Tomaselli S., Guerrini R., Salvadori S., D’Ursi A. M., Temussi P. A., Picone D. (2002). Solution structure of the Alzheimer's amyloid beta-peptide (1–42) in an apolar microenvironment. Similarity with a virus fusion domain. Eur. J. Biochem..

[16] Zagorski M. G., Colin J. B. (1992). NMR Studies of Amyloid beta- peptides: proton assignments, secondary structure, and mechanism of an alpha-helix–beta-sheet conversion for a homologous, 28-residue, N-terminal fragment. Biochemistry.

[17] Tomaselli S., Esposito V., Vangone P., Nico A. J., Bonvin Alexandre M. J. J., Guerrini R., Tancredi T., Temussi P. A., Picone D. (2006). The alpha-to-beta conformational transition of Alzheimer's Abeta-(1–42) peptide in aqueous media is reversible: a step by step conformational analysis suggests the location of beta conformation seeding. Chembiochem.

[18] Fraga A. S., Esteves A. C., Micaelo N., Cruz P. F., Brito R. M., Nutley M., Cooper A., Barros M. M., Pires E. M. (2012). Functional and conformational changes in the aspartic protease cardosin A induced by TFE. Int. J. Biol. Macromol..

[19] Vivekanandan S., Brender J. R., Lee S. Y., Ramamoorthy A. (2011). A partially folded structure of amyloid-beta (1–40) in an aqueous environment. Biochem. Biophys. Res. Commun..

[20] Usachev K. S., Filippov A. V., Antzutkin O. N., Klochkov V. V. (2013). Use of a combination of the RDC method and NOESY NMR spectroscopy to determine the structure of Alzheimer's amyloid Ab10-35 peptide in solution and in SDS micelles. Eur. Biophys. J..

[21] Keeler J., Freeman R., Shaka A. J. (1983). Evaluation of a new broadband decoupling sequence: WALTZ-16. J. Magn. Reson..

[22] Cavanagh J., Rance M. (1990). Sensitivity improvement in isotropic mixing (TOCSY) experiments. J. Magn. Reson..

[23] Neuhaus D., Williamson M. P. (1989). The nuclear Overhauser Effect in Structural and Conformational Analysis, 2^nd^ Edition.

[24] Piotto M., Saudek V., Sklenár V. (1992). Gradient-tailored excitation for single-quantum NMR spectroscopy of aqueous solutions. J. Biomol. NMR.

[25] Leppik R., Piotto M., Saudek V., Sklenar V. (1993). Gradient-Tailored Water Suppression for 1H-15N HSQC experiments optimized to retain full sensitivity. J. Magn. Reson. Ser. A..

[26] Brünger A. T., Adams P. D., Clore G. M., DeLano W. L., Gros P., Grosse-Kunstleve R. W., Jiang J. S., Kuszewski J., Nilges M., Pannu N. S. (1998). Crystallography & NMR system: a new software suite for macromolecular structure determination. Acta Crystallogr. D, Biol. Crystallogr..

[27] Chou P., Fasmanm G. (1974). Prediction of protein conformation. Biochemistry.

[28] Petersen B., Petersen T. N., Andersen P., Nielsen M., Lundegaard C. (2009). A generic method for assignment of reliability scores applied to solvent accessibility predictions. BMC Struct. Biol..

[29] Cole C., Barber J. D., Barton G. J. (2008). The Jpred 3 secondary structure prediction server. Nucleic Acids Res..

[30] Zhang Y. (2008). I-TASSER server for protein 3D structure prediction. BMC Bioinformatics..

[31] Creighton T. E. (1993). Proteins: Structures and Molecular Properties, 2^nd^ Edition.

[32] Murphy M. P., LeVine H. (2010). Alzheimer's disease and the amyloid-beta peptide. J. Alzheimers Dis..

[33] Cerf E., Sarroukh R., Tamamizu-Kato S., Breydo L., Derclaye S., Dufrêne Y. F., Narayanaswami V., Goormaghtigh E., Ruysschaert J-M., Raussens V. (2009). Antiparallel beta-sheet: a signature structure of the oligomeric amyloid beta-peptide. Biochem. J..

[34] Li L., Darden T. A., Bartolotti L., Kominos D., Pedersen L. G. (1999). An atomic model for the pleated beta-sheet structure of Abeta amyloid protofilaments. Biophys. J..

[35] Hoyer W., Grönwall C., Jonsson A., Ståhl S., Härd T. (2008). Stabilization of a beta-hairpin in monomeric Alzheimer's amyloid-beta peptide inhibits amyloid formation. Proc. Natl. Acad. Sci. U. S. A..

[36] Wang H. Y., Lee D. H., D’Andrea M. R., Peterson P. A., Shank R. P., Reitz A. B. (2000). beta-Amyloid (1–42) binds to alpha7 nicotinic acetylcholine receptor with high affinity. Implications for Alzheimer's disease pathology. J. Biol. Chem..

[37] Maatuk N., Samson A. O. (2012). Modeling the binding mechanism of Alzheimer's Aβ1–A42 to nicotinic acetylcholine receptors based on similarity with snake α-neurotoxins. Neurotoxicology.

